# Differences in risk factors for 3 types of stroke: UK prospective study and meta-analyses

**DOI:** 10.1212/WNL.0000000000004856

**Published:** 2018-01-23

**Authors:** Alison J. Price, F. Lucy Wright, Jane Green, Angela Balkwill, Sau Wan Kan, TienYu Owen Yang, Sarah Floud, Mary E. Kroll, Rachel Simpson, Cathie L.M. Sudlow, Valerie Beral, Gillian K. Reeves

**Affiliations:** From the Nuffield Department of Population Health (A.J.P., F.L.W., J.G., A.B., S.W.K., T.Y.O.Y., S.F., R.S., V.B., G.K.R.) and National Perinatal Epidemiology Unit (M.E.K.), University of Oxford; London School of Hygiene and Tropical Medicine (A.J.P.); and Centre for Clinical Brain Science (C.L.M.S.), University of Edinburgh, UK.

## Abstract

**Objective:**

To compare associations of behavioral and related factors for incident subarachnoid hemorrhage and intracerebral hemorrhage and ischemic stroke.

**Methods:**

A total of 712,433 Million Women Study participants without prior stroke, heart disease, or cancer reported behavioral and related factors at baseline (1999–2007) and were followed up by record linkage to national hospital admission and death databases. Cox regression yielded adjusted relative risks (RRs) by type of stroke. Heterogeneity was assessed with χ^2^ tests. When appropriate, meta-analyses were done of published prospective studies.

**Results:**

After 12.9 (SD 2.6) years of follow-up, 8,128 women had an incident ischemic stroke, 2,032 had intracerebral hemorrhage, and 1,536 had subarachnoid hemorrhage. In women with diabetes mellitus, the risk of ischemic stroke was substantially increased (RR 2.01, 95% confidence interval [CI] 1.84–2.20), risk of intracerebral hemorrhage was increased slightly (RR 1.31, 95% CI 1.04–1.65), but risk of subarachnoid hemorrhage was reduced (RR 0.43, 95% CI 0.26–0.69) (heterogeneity by stroke type, *p* < 0.0001). Stroke incidence was greater in women who rated their health as poor/fair compared to those who rated their health as excellent/good (RR 1.36, 95% CI 1.30–1.42). Among 565,850 women who rated their heath as excellent/good, current smokers were at an increased risk of all 3 stroke types, (although greater for subarachnoid hemorrhage [≥15 cigarettes/d vs never smoker, RR 4.75, 95% CI 4.12–5.47] than for intracerebral hemorrhage [RR 2.30, 95% CI 1.94–2.72] or ischemic stroke [RR 2.50, 95% CI 2.29–2.72]; heterogeneity *p* < 0.0001). Obesity was associated with an increased risk of ischemic stroke and a decreased risk of hemorrhagic stroke (heterogeneity *p* < 0.0001). Meta-analyses confirmed the associations and the heterogeneity across the 3 types of stroke.

**Conclusion:**

Classic risk factors for stroke have considerably different effects on the 3 main pathologic types of stroke.

Stroke is a leading cause of death and disability worldwide.^1–3^ Heart disease, hypertension, and diabetes mellitus, as well as behavioral and related factors such as smoking and obesity, are known to be associated with greater overall risk of stroke.^4^ The 3 main pathologic types of stroke are ischemic stroke, intracerebral hemorrhage, and subarachnoid hemorrhage, but evidence on the extent to which risk factors differ for these 3 different types is limited. Few studies have been sufficiently large to compare reliably the risk factors for the 3 types of stroke, and there is the potential problem of reverse causation bias whereby behavior changes caused by prior vascular disease or general poor health can distort associations between health-related behaviors and stroke.

We used data from a large prospective study of UK women to compare risk factors for ischemic stroke, intracerebral hemorrhage, and subarachnoid hemorrhage. To minimize biases associated with reverse causation, we excluded women with prior vascular disease or cancer from all analyses and, in some analyses, excluded women who rated their health as poor or fair and excluded the first 3 years of follow-up. To set our key findings in context, we reviewed the published evidence and, when appropriate, performed meta-analyses combining our results with those from previously published prospective studies.

## Methods

### Study population

Between 1996 and 2001, 1.3 million women joined the UK Million Women Study by completing a recruitment questionnaire on sociodemographic, health, and lifestyle characteristics.^5^ Study participants have been sent resurvey questionnaires every 3 to 5 years. Study questionnaires and details of the data and access policies can be viewed on the study website.^6^

### Standard protocol approvals, registrations, and patient consents

The Multi-Centre Research Ethics Committee for Anglia and Oxford approved the study (REC reference 97/5/001). Participants provided written consent to follow-up through medical records.

### Exposures

Baseline for these analyses was the 3-year resurvey questionnaire (completed on average 3.3 [SD 1.1] years after recruitment), which was the first time that participants were asked to rate their overall health (as excellent, good, fair, or poor), an important predictor of stroke risk.^7^ Women reported a history of diabetes mellitus and hypertension and whether they were being treated for it, heart disease, and other conditions, as well as current smoking, alcohol consumption, weight, and physical activity. Baseline information was used, except for region of residence, socioeconomic status, educational attainment, and height, which were recorded only at recruitment. Reported levels of physical activity were converted to excess metabolic equivalents.^8^ Because we could not distinguish between never and former drinkers, alcohol-associated risks were assessed in current drinkers at baseline.

### Outcomes

Follow-up of all women in the cohort for deaths, emigration, and hospital admissions (inpatient and day case) was by electronic record linkage to routinely collected National Health Service (NHS) databases using each individual's NHS identification number and date of birth. Linked data were provided by NHS Digital (England) and NHS Information Services Division (Scotland). The data provided included date for each event and cause, coded to the ICD-10.

We defined first stroke as the earliest hospital admission with stroke, or death with stroke certified as the underlying cause, that occurred after baseline. Stroke outcome included any diagnosis of a specific stroke type: subarachnoid hemorrhage (ICD-10 code I60), intracerebral hemorrhage (ICD-10 code I61), and ischemic stroke (ICD-10 code I63). Women with >1 type of stroke at the first hospital admission (n = 325) or with an unspecified type only (code I64, n = 2,443) were censored on that date.

### Statistical analysis

Cox regression models were used to estimate hazard ratios, henceforth referred to as relative risks (RRs), and their 95% confidence intervals (CIs), for incident stroke in relation to self-rated health, reported treatment for diabetes mellitus and hypertension, smoking status, alcohol consumption, body mass index, and physical activity. When >2 groups were compared, group-specific CIs were calculated using the variance of the log risk for each group.^9^

Women were excluded from the analysis in this order: if at baseline they did not report self-rated health (n = 25,682); if they had a hospital record of cerebrovascular disease (n = 4,027) or ischemic heart disease (n = 17,002); if they had reported a prior stroke or transient ischemic attack (n = 10,064) or prior heart disease (n = 37,628); if they had a prior cancer registration other than nonmelanoma skin cancer (n = 16,386); and if they had completed the baseline survey but were no longer registered with the NHS (n = 105). After these exclusions, 712,433 women remained for analysis.

Person-years were calculated from the date the baseline questionnaire was completed to first hospital record of any stroke (ICD codes I60–I64), death, emigration, or March 31, 2015 (the latest date when complete information was available), whichever was earliest. Time in study was the underlying time variable, and analyses were stratified by year of birth (1930 or before, 1931 to 1949 in single years, 1950 or later) and of completion of the baseline questionnaire (2000 or before, 2001, 2002, 2003, 2004 or later) and adjusted for region of residence (10 geographical areas in the United Kingdom), socioeconomic status (5 levels of the Townsend index^10^), educational attainment (tertiary, secondary/technical, no formal qualifications^11^), and when appropriate for smoking (never, past, current <5, 5–9, 10–14, 15–19, 20–24, ≥25 cigarettes/d), alcohol consumption (none, ≤6, 7–14, ≥15 drinks/wk), body mass index (<25, 25–29.9, ≥30 kg/m^2^), physical activity (thirds of excess metabolic equivalents), and menopausal hormone therapy (never, past, current). For adjustment variables, missing values were assigned to an unknown category and represented <5% of the data in every variable. Sensitivity analyses excluding the first 3 years of follow-up were conducted.

We estimated log-linear trends in risk, when appropriate, scoring each category as the mean within-category measure. To allow for measurement error, regression dilution, and changes over time, estimates of trend in risk for tobacco and alcohol consumption, body mass index, and physical activity used mean remeasured values for the baseline categories being compared (appendix e-1, links.lww.com/WNL/A63). We used χ^2^ tests to assess heterogeneity across stroke types in the trend estimates. Statistical tests were 2 sided. Analyses used Stata version 14.0 (StataCorp, College Station, TX).^12^

### Systematic review and meta-analysis

Recent reviews and meta-analyses have compared associations across the 3 stroke types with body mass index,^13^ hypertension,^14,15^ physical activity,^16^ and alcohol^17^ and were not repeated here. We focused on diabetes mellitus and smoking, for which there had not been a recent relevant meta-analysis (appendix e-2, links.lww.com/WNL/A63, and figures e-1 and e-2, links.lww.com/WNL/A61, and tables e-1 and e-2, links.lww.com/WNL/A62, give search strategy and further details). Prospective studies were eligible for inclusion if they reported risk estimates for all of the 3 stroke types by diabetes mellitus or smoking in the same report, using the same analytic method for each type. Because the full consequences of the smoking epidemic in Asia have not yet emerged,^18^ studies were grouped into those from Asia and those from Europe or North America. We combined type-specific stroke estimates across studies using inverse variance-weighted methods and tested heterogeneity across stroke types by χ^2^ tests.

## Results

A total of 712,433 women without prior stroke, heart disease, or cancer and with information on self-rated health were followed up for 12.9 (SD 2.6) years on average. Their mean age at baseline was 59.8 (SD 4.9) years. There were large differences in behavioral and health characteristics in relation to self-rated health status (table e-3, links.lww.com/WNL/A62). Compared to women with excellent/good self-rated health, those with poor/fair self-rated health were more likely to be of lower socioeconomic status, obese, nondrinkers of alcohol, current smokers, and currently treated for diabetes mellitus or hypertension.

During follow-up, 11,696 women were admitted to hospital or died with a specified type of stroke: 8,128 had a first ischemic stroke, 2,032 had a first intracerebral hemorrhage, and 1,536 had a first subarachnoid hemorrhage. For only 4% (460 of 11,696), the first record of a stroke was the underlying cause of death. Stroke was listed as the primary diagnosis in >90% of the hospital admissions.

**Figure 1:**
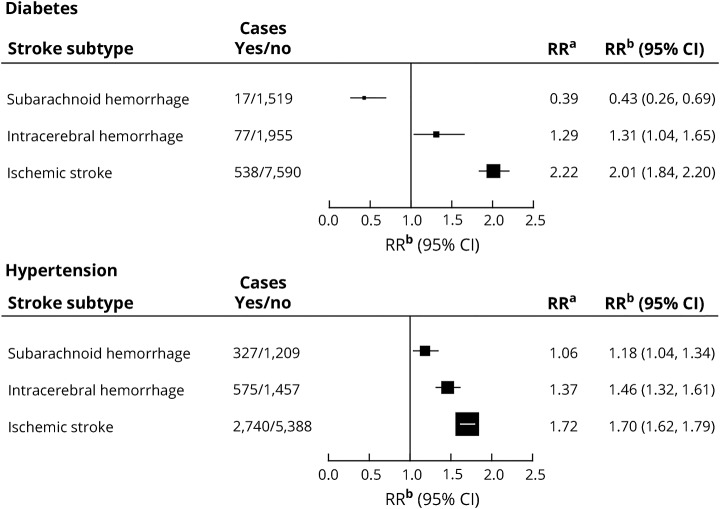
Relative risk of stroke associated with diabetes mellitus and hypertension requiring treatment, reported at baseline RR^a^ is the relative risk for diabetes mellitus/hypertension compared to no diabetes mellitus/hypertension stratified by year of birth and calendar year at baseline and adjusted for region of residence, educational attainment, and socioeconomic status. RR^b^ is the relative risk for diabetes mellitus/hypertension compared to no diabetes mellitus/hypertension, as for RR^a^, and additionally adjusted for use of menopausal hormones, smoking, alcohol consumption, body mass index, and physical activity. CI = confidence interval.

Among women reporting at baseline that they had diabetes mellitus that required treatment, compared to other women, the RR (adjusted for region of residence, deprivation, education, smoking, alcohol use, body mass index, physical activity and menopausal hormone therapy) was substantially increased for ischemic stroke (RR 2.01, 95% CI 1.84–2.20), slightly but significantly increased for intracerebral hemorrhage (RR 1.31, 95% CI 1.04–1.65), and substantially reduced for subarachnoid hemorrhage (RR 0.43, 95% CI 0.26–0.69) (test for heterogeneity between the 3 stroke types *p* < 0.0001). The exclusion of body mass index from the fully adjusted model for subarachnoid hemorrhage had a minimal effect (the diabetes mellitus–associated RR declined from 0.43 to 0.40). Among women who reported a history of hypertension requiring treatment, there was an increased risk of each stroke type: ischemic stroke (RR 1.70, 95% CI 1.62–1.79), intracerebral hemorrhage (RR 1.46, 95% CI 1.32–1.61), and subarachnoid hemorrhage (RR 1.18, 95% CI 1.04–1.34), with significant heterogeneity by type (*p* < 0.0001; figure 1).

In analyses adjusted for diabetes mellitus and hypertension at baseline and for region of residence, deprivation, education, smoking, alcohol use, body mass index, and physical activity, risk of stroke was significantly greater in women who rated their health as poor/fair than in those who rated their health as excellent/good (RR 1.36, 95% CI 1.30–1.42). To minimize the potential for reverse causation bias resulting from changes in behavior due to preexisting ill health, analyses of associations of behavioral and related factors with risk of stroke were restricted to the 565,850 women who rated their health at baseline as excellent or good.

**Figure 2:**
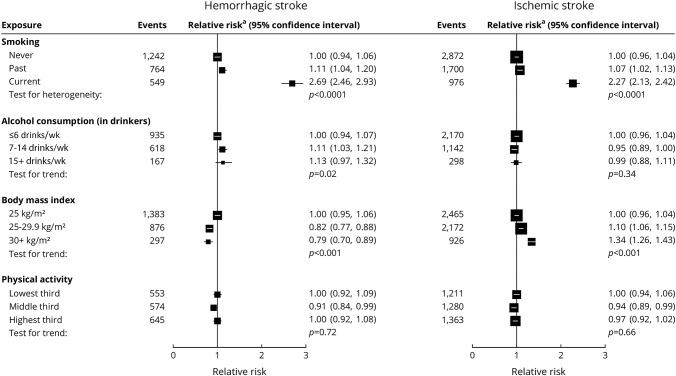
Relative risk of hemorrhagic and ischemic stroke associated with classic behavior-related vascular risk factors at baseline in those with excellent/good self-rated health Relative risk^a^ is stratified by year of birth and calendar year at baseline and adjusted for region of residence, educational attainment, socioeconomic status, and use of menopausal hormones, and, when appropriate, for smoking, alcohol consumption, body mass index, and physical activity. Test for heterogeneity in the relative risk for current vs never smoking between hemorrhagic and ischemic stroke: *p* = 0.01. Test for heterogeneity in trend between hemorrhagic and ischemic stroke: alcohol consumption *p* = 0.02, body mass index *p* < 0.0001, and physical activity *p* = 0.59.

**Figure 3:**
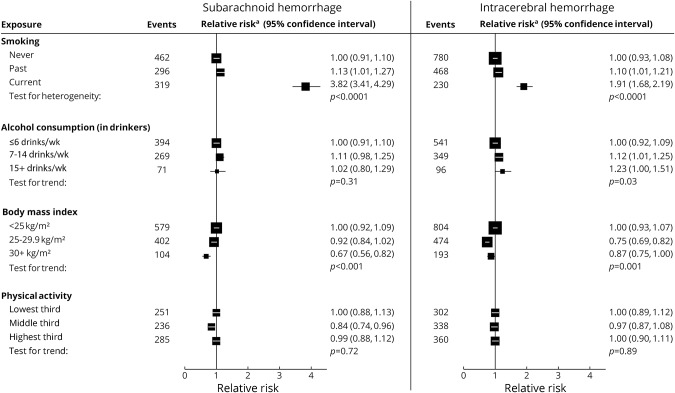
Relative risk of subarachnoid and intracerebral hemorrhage associated with classic behavior-related factors at baseline in those with excellent/good self-rated health Relative risk^a^ is stratified by year of birth and calendar year at baseline and adjusted for region of residence, educational attainment, socioeconomic status, and use of menopausal hormones, and, when appropriate, for smoking, alcohol consumption, body mass index, and physical activity. Test for heterogeneity in the relative risk for current vs never smoking between subarachnoid hemorrhage and intracerebral hemorrhage: *p* < 0.0001. Test for heterogeneity in trend between subarachnoid hemorrhage and intracerebral hemorrhage: alcohol consumption *p* = 0.51, body mass index *p* = 0.54, and physical activity *p* = 0.86.

**Figure 4:**
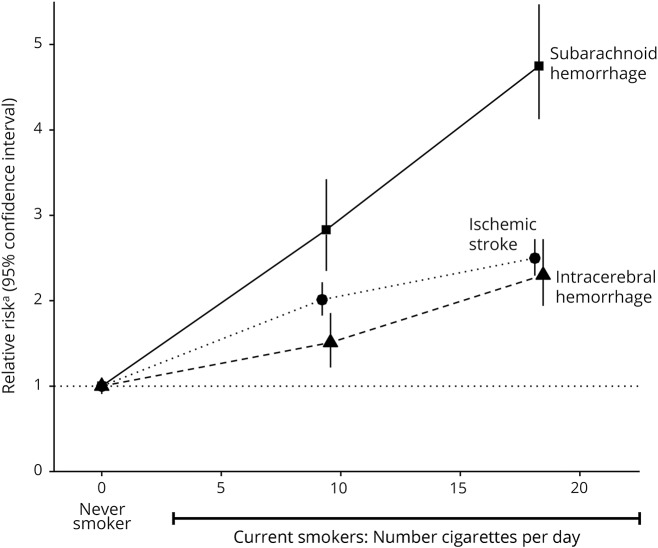
Relative risk of stroke type by amount smoked in current smokers at baseline in those with excellent/good self-rated health Relative risk^a^ is the relative risk in current vs never smokers stratified by year of birth and calendar year at baseline and adjusted for region of residence, educational attainment, socioeconomic status, use of menopausal hormones, alcohol consumption, body mass index, and physical activity.

In women with excellent/good self-rated health, current smoking was associated with an increased risk of both hemorrhagic and ischemic stroke (figure 2), with risk greater for subarachnoid than for intracerebral hemorrhage (figure 3). The associations are dose dependent (figure 4), with RRs for ≥15 cigarettes/d being 4.75 (95% CI 4.12–5.47) for subarachnoid hemorrhage, 2.30 (95% CI 1.94–2.72) for intracerebral hemorrhage, and 2.50 (95% CI 2.29–2.72) for ischemic stroke (heterogeneity across stroke types *p* < 0.0001; figure 4).

Adiposity was associated with an increased risk of ischemic stroke but reduced risk of hemorrhagic stroke, with no significant difference in the associations between subarachnoid hemorrhage and intracerebral hemorrhage.

Alcohol consumption in drinkers showed little association with either ischemic or hemorrhagic stroke, although there was some suggestion for increased risk of intracerebral hemorrhage with higher alcohol consumption. There was no significant association between physical activity and risk for any stroke type.

In analyses of women reporting good/excellent self-rated health, exclusion of the first 3 years of follow-up had little effect on the findings for any of the risk factors considered (figure e-3, links.lww.com/WNL/A61).

Results corresponding to figures 2 and 3 for women rating their health as poor/fair at baseline are shown in figures e-4 and e-5, links.lww.com/WNL/A61. In women with poor/fair health, certain associations differed from those in women with excellent/good health in ways that were consistent with poor health having changed behavior. In particular, associations with smoking were considerably attenuated, and associations with physical activity were qualitatively different.

### Systematic review and meta-analysis

**Figure 5:**
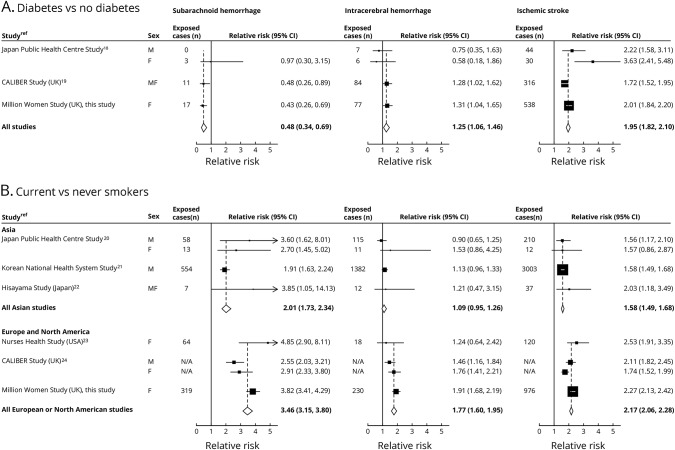
Meta-analysis of prospective studies that reported the risk of the 3 types of stroke (A) Diabetes mellitus vs no diabetes mellitus (heterogeneity between stroke types *p* < 0.001) and (B) current vs never smokers (heterogeneity between stroke types *p* < 0.001 in Asian studies and *p* < 0.001 in European and North American studies). CI = confidence interval; RR = relative risk.

For diabetes mellitus, we identified 2 eligible studies^18,19^ (figure 5A). Combined results from 3 studies (including ours) showed about a halving in the diabetes mellitus–associated RR of subarachnoid hemorrhage (RR 0.48, 95% CI 0.34–0.69), a modest increase in risk of intracerebral hemorrhage (RR 1.25, 95% CI 1.06–1.46), and almost a doubling in risk of ischemic stroke (RR 1.95, 95% CI 1.82–2.10; test for heterogeneity across the 3 types *p* < 0.001).

For current smoking, 5 previously published studies were identified, 3 from Asia^19–21^ and 2 from Europe or North America.^22,23^ Combined RRs from the 6 studies (including ours) were increased for all stroke types (figure 5B). The RRs associated with current smoking were consistently higher in Europe/North America than in Asia, but in both regions, current smokers were at greater risk for subarachnoid hemorrhage (RR 3.46 and 2.01 for Europe/North America and for Asia, respectively) than for intracerebral hemorrhage (RR 1.77 and 1.09, respectively) or for ischemic stroke (RR 2.17 and 1.58, respectively; test for heterogeneity across the 3 types *p* < 0.001 for each region).

## Discussion

In this large prospective epidemiologic study with almost 12,000 incident strokes of the 3 main pathologic types, we found considerable heterogeneity in the risks of subarachnoid hemorrhage, intracerebral hemorrhage, and ischemic stroke associated with a history of diabetes mellitus, a history of hypertension, current smoking, and adiposity. Physical activity and alcohol consumption were not strongly related to risk of any of the 3 types of stroke.

A meta-analysis of results from this and previously published prospective studies showed about a halving in the risk of subarachnoid hemorrhage associated with a history of diabetes mellitus. In contrast, there was a small increase in risk of intracerebral hemorrhage and a doubling in risk of ischemic stroke. The increased risk of ischemic stroke in people with diabetes mellitus is well established and believed to have a mechanism similar to that for ischemic heart disease.^24^ A reduced risk of subarachnoid hemorrhage associated with diabetes mellitus has been reported in several retrospective and prospective studies,^25^ but few prospective studies have directly compared diabetes mellitus–associated risk for subarachnoid hemorrhage with the risk for the other types of stroke.^26,27^ The mechanism for the observed reduction in diabetes mellitus–associated risk for subarachnoid hemorrhage is not clear.

In the interpretation of our findings on risk of stroke in relation to hypertension, it is important to note that our analyses were necessarily based on whether a woman reported having hypertension that required treatment at baseline because information on blood pressure was not generally available. Most previous studies that have examined the relationship between hypertension and stroke risk have used measured blood pressure as the exposure,^13,14,27^ often reporting stronger relationships for hemorrhagic than ischemic stroke.^15,28^ The extent to which our findings for a history of hypertension are relevant to the corresponding pattern in relation to measured blood pressure is uncertain.

The risk of each of the 3 stroke types was increased in current smokers, and the relationships were dose dependent. In every study in our meta-analysis, the magnitude of the smoking-associated risk was greater for subarachnoid hemorrhage than for intracerebral hemorrhage or ischemic stroke. These results are consistent with those from meta-analyses restricted to subarachnoid hemorrhage alone.^25,29^ The distribution of stroke types is known to differ between populations of Asian and European origin,^30^ with hemorrhagic strokes making up a greater proportion of all strokes in Asia. The differences in smoking-related risks seen here between studies from Europe and North America and those from Asia may reflect the fact that the full health consequences of the smoking epidemic in Asia have not yet emerged.^18^ Likewise, any apparent differences by sex in smoking-related risks of stroke reported in the past may reflect the fact that the full health consequences of the smoking epidemic emerged later in women than men; in the 21st century, the effects of smoking are similar in men and women.^31^

For obesity, we previously reported an increased risk of ischemic stroke, and decreased risk of both types of hemorrhagic stroke, which is confirmed with updated data here, consistent with the meta-analysis of results from published prospective studies.^13^

Some have reported a reduced risk of ischemic and hemorrhagic stroke associated with moderate to vigorous physical activity^8,16^ but did not take previous ill health into account. Any apparent increase in stroke risk associated with inactivity may be due to reverse causation, with women reducing their activity because of poor health, which itself is associated with an increased risk of stroke. Our finding of an apparently lower risk of stroke with greater physical activity only in women rating their health as poor or fair at baseline supports this view.

Levels of alcohol consumption in our participants are relatively low, and we found limited evidence for an association between amount consumed and stroke risk, although a modestly increased risk was observed for intracerebral hemorrhage with higher alcohol consumption. We did not include nondrinkers in our trend tests because we cannot distinguish between never drinkers and former drinkers. Drinking no alcohol was reported much more commonly in those with poor/fair health than in those with excellent/good health because some may have reduced their consumption because of ill health. A recent review and meta-analysis^17^ found modestly increased risks for each stroke type in heavy drinkers.

Strengths of the Million Women Study include its prospective design, large size, and virtually complete long-term follow-up by record linkage to routinely collected NHS databases. Only ≈1% of participants were lost to follow-up, largely because of emigration, but they were included in the analyses up to the date when they were lost. We found full agreement with primary care records for 96% of hospital admissions for subarachnoid hemorrhage, 78% for intracerebral hemorrhage, and 86% for cerebral ischaemia.^32^

By excluding women with prior stroke, ischemic heart disease, or cancer, and those with self-rated poor/fair health at baseline, we minimized the potential bias associated with reverse causation for health-related behavioral factors. To account for measurement error, regression dilution, and changes over time, we used remeasured values of these factors in the estimation of trends in risk.

Our analyses were confined to strokes that resulted in hospital admission. Because hemorrhagic strokes are more likely than ischemic strokes to result in a hospital admission probably because of their relative severity, hemorrhagic stroke may well be overrepresented here compared with the general population. This should not, however, bias findings for stroke types. When we compared primary care and hospital admission data for a subset of the cohort, only 6 of 864 participants without a hospital admission for stroke had any record of a cerebrovascular event in their primary care records.^32^

Although we adjusted for age, socioeconomic status, and lifestyle factors, we cannot rule out residual confounding by unmeasured factors. The measures available to us for hypertension were limited and make it difficult to interpret our findings. We studied UK women only, but our meta-analyses, which found similar results, included men and populations from other regions of the world.

Taken together, results from this large cohort of UK women and from meta-analyses show substantial heterogeneity in the associations of diabetes mellitus, smoking, and adiposity with incident subarachnoid hemorrhage, intracerebral hemorrhage, and ischemic stroke. These findings confirm that the 3 main pathologic types of stroke have considerably different etiologies.

## Glossary

CI: confidence interval
ICD-10: *International Classification of Diseases, 10th revision*
NHS: National Health Service
RR: relative risk
